# A polymerase III-like reinitiation mechanism is operating in regulation of histone expression in archaea

**DOI:** 10.1111/j.1365-2958.2007.06084.x

**Published:** 2008-03

**Authors:** Patrizia Spitalny, Michael Thomm

**Affiliations:** Department of Microbiology, University of Regensburg Universitätsstraße 31, 93053 Regensburg, Germany

## Abstract

An archaeal histone gene from the hyperthermophile *Pyrococcus furiosus* containing four consecutive putative oligo-dT terminator sequences was used as a model system to investigate termination signals and the mechanism of termination *in vitro*. The archaeal RNA polymerase terminated with high efficiency at the first terminator at 90°C when it contained five to six T residues, at 80°C readthrough was significantly increased. A putative hairpin structure upstream of the first terminator had no effect on termination efficiency. Template competition experiments starting with RNA polymerase molecules engaged in ternary complexes revealed recycling of RNA polymerase from the terminator to the promoter of the same template. This facilitated reinitiation was dependent upon the presence of a terminator sequence suggesting that pausing at the terminator is required for recycling as in the RNA polymerase III system. Replacement of the sequences immediately downstream of the oligo-dT terminator by an AT-rich segment improved termination efficiency. Both AT-rich and GC-rich downstream sequences seemed to impair the facilitated reinitiation pathway. Our data suggest that recycling is dependent on a subtle interplay of pausing of RNA polymerase at the terminator and RNA polymerase translocation beyond the oligo-dT termination signal that is dramatically affected by downstream sequences.

## Introduction

The mechanism of initiation of transcription in archaea is RNA polymerase II (pol II)-like (Bell and Jackson, 2001; Geiduschek and Ouhammouch, 2005; Thomm, 2007) and is dependent upon the general transcription factors TATA-binding protein (TBP) and transcription factor B (TFB), both related in structure and function to eukaryotic TBP and transcription factor IIB (TFIIB). Recent evidence suggests that a third factor, TFE that is homologous to the N-terminal part of the α subunit of TFIIE plays in addition a pivotal role in initiation (Bell *et al*., 2001; Hanzelka *et al*., 2001; Werner and Weinzierl, 2005; Naji *et al*., 2007) and also in elongation by stabilizing the open complex and transcription bubble (Grünberg *et al*., 2007). Archaeal transcriptional terminators were early described to contain oligo-T stretches (Reiter *et al*., 1988; Brown *et al*., 1989; Thomm *et al*., 1994) that are also recognized by RNA pol III as terminator signals (Geiduschek and Kassavetis, 1992; Gunnery *et al*., 1999; Braglia *et al*., 2005), and a detailed study using a single-round *in vitro* system from a thermophilic archaeon has shown that these oligo-dT sequences without preceding RNA hairpin structures are sufficient to direct termination by an archaeal RNA polymerase (RNAP) *in vitro* (Santangelo and Reeve, 2006). Unlike in pol I and pol II transcripts, the 3′ ends of pol III and of archaeal transcripts are generated by transcriptional termination. Thus, both the sequences of terminators and the general mechanism of archaeal transcription termination seem to be pol III-like. However, the finding that rho-independent bacterial terminators and the bacterial rho-factor can mediate termination of transcription in an archaeal system (Santangelo and Reeve, 2006) show also some superficial similarities of archaeal termination to termination in the bacterial system.

With one exception (Thomm *et al*., 1994), the complete transcription cycle involving initiation elongation and termination on an intact and complete archaeal gene has not yet been studied. Such an investigation is likely to lead to important insights, because the mechanism of transcription reinitiation involving direct recycling of RNAP from terminator to promoter represents an important aspect of gene regulation in particular in the pol III, but also in other transcription systems (Dieci and Sentenac, 1996; 2003). Furthermore, the sequences immediately downstream of terminator sequences have been shown to be involved in pausing (Lee *et al*., 1990; Palangat *et al*., 2004), which is a precursor of transcriptional arrest and termination, and the effects of downstream sequences on termination and recycling of RNAP have not yet been studied in the archaeal system.

Using a complete gene encoding an archaeal histone as template, we demonstrate in a hyperthermophilic system that reinitiation has been established as regulatory mechanisms in the archaeal transcriptional machinery which is thought to be the evolutionary precursor of the eukaryotic system. In addition, we show that GC-rich sequences downstream of the terminator inhibit recycling of RNAP from the terminator to promoter, and are therefore likely to reduce the levels of gene expression at high temperatures.

## Results

### Transcriptional termination at 90°C

**Fig. 1:**
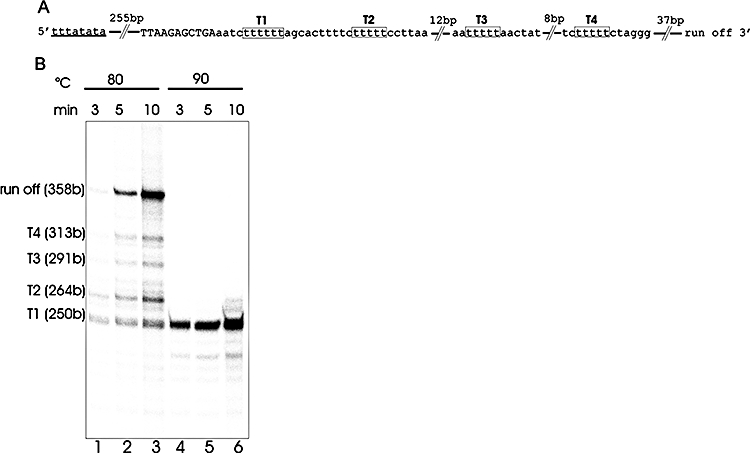
Termination efficiency is increased at 90°C. In (A) the sequence of the RNA-like strand of *hpyA1* cloned into pUC19 is depicted from the TATA box (underlined) to the downstream end created by PstI digestion. The part of the sequence belonging to the ORF is written in capital letters. The main termination site (T1) and the tree back-up terminators (T2–T4) are boxed. *In vitro* transcription of the template displayed in (A) at 80°C and 90°C, respectively, is shown in (B). The lengths of the transcripts are indicated on the left. Transcription assays were performed with 46 nM RNAP, 238 nM TBP and 147 nM TFB.

**Fig. 5:**
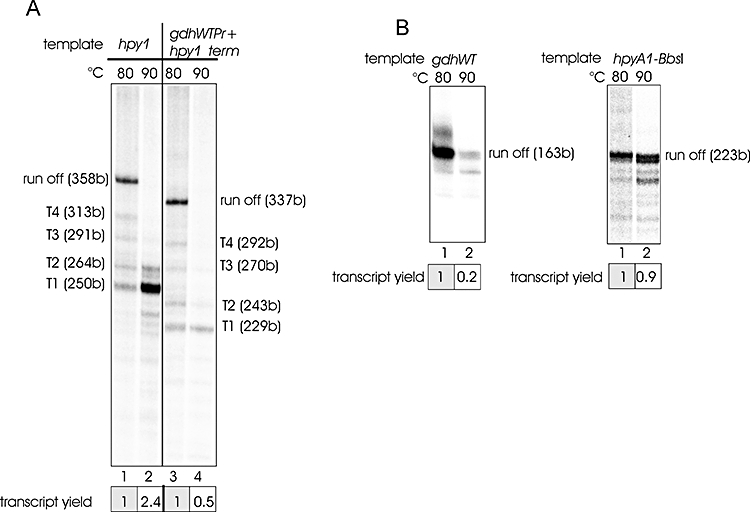
Both terminator and associated promoter are necessary for high transcription rate at 90°C. A. The *hpyA1* terminator region was fused to the promoter region of *gdh* followed by 163 bp of *gdh* sequence. The WT and the fused DNA template were incubated 10 min at the temperatures indicated on top of the lanes. The RNA products were analysed on 6% PA gels. The transcriptional activity at 90°C, relative to the activity at 80°C which was defined as 1, is indicated below the lanes. The values for templates carrying the *hpyA*1 promoter were confirmed by four independent experiments. B, Run-off transcripts from a DNA fragment carrying the *gdh* promoter but lacking the *hypA1* terminator, and from the truncated *hpyA1* template (template 3 of Fig. 4E) lacking the terminator region after restriction with BbsI. Transcription assays were performed with 46 nM RNAP, 238 nM TBP and 147 nM TFB.

The histone gene *hpyA1* from *Pyrococcus furiosus* was chosen for termination experiments, because it shows four consecutive oligo-dT sequences (T1 to T4) directly following the open reading frame (ORF) (Fig. 1A) suspected to act as termination sites. The little information available on archaeal termination suggests that mechanisms that are more closely related to pol III than to bacterial RNAP. But still, archaeal termination is poorly little understood, especially in hyperthermophilic archaea. To investigate the termination events in hyperthermophilic archaea, the histone-encoding gene *hpyA1* was amplified from *P. furiosus* genomic DNA and cloned into a plasmid vector. It shows a high transcription rate in *in vitro* transcription assays (Fig. 1B). It is a short protein encoding gene which can easily be transcribed completely in multiple round *in vitro* assays from the start point to the termination sites. Incubation of this template in linearized state at 80°C led to readthrough events at every termination site resulting in a predominant run-off transcript (358 b). In contrast, when incubated at 90°C, a temperature more close to the growth optimum of *P. furiosus*, the first terminator was recognized very efficiently (Fig. 1B), leading to a predominant transcript of 250 nt (Fig. 1B, lanes 4–6). When truncated, linearized templates (not containing a terminator) were used in *in vitro* transcription experiments; template activity at 90°C was significantly lower [for *hpyA1* see Fig. 5B, right panel; for a comparison of the template activity of *Pyrococcus glutamate deydrogenase* (*gdh*) promoter at 80 and 90°C, see Fig. 5B, left panel]. Under our *in vitro* conditions, purified RNA subjected to an incubation temperature of 90°C is degraded with a half-life time of ∼20 min (Hethke, 1997; Hethke *et al*., 1999). Therefore, a specific terminator-dependent mechanism must exist allowing the high expression rate of the *hpyA1* gene *in vitro.*

### The minimal termination signal is T_5_

As shown in Fig. 1, efficient archaeal termination at least at the *hpyA1* terminator is mediated by the archaeal RNAP alone without the need for additional factors. A simple run of T residues at the end of the *hpyA1* gene serves as termination signal for the archaeal RNAP. In bacteria, usually seven to nine T residues following a dyad symmetry element facilitate factor-independent termination. The minimal termination signal recognized by eukaryal pol III varies among different species (Bogenhagen and Brown, 1981; Cozzarelli *et al*., 1983; Allison and Hall, 1985; Hamada *et al*., 2000). The T tract leads to extensive pausing of pol III (Matsuzaki *et al*., 1994; Yin *et al*., 1999), and termination efficiency tends to increase with the length of the T cluster (Allison and Hall, 1985).

**Fig. 2:**
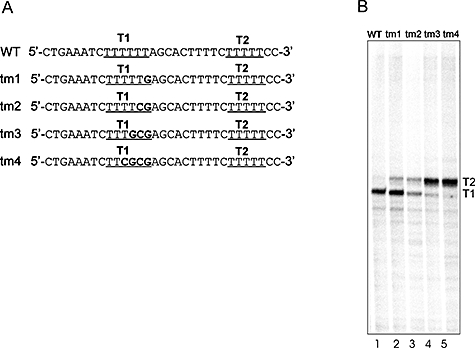
The minimal termination signal is T_5_. A. Point mutations (bold) altering the length of the T tract were introduced into the 3′ flanking region of termination site T1. The effect of the different 3′ mutations is shown in (B). *In vitro* transcription experiments were performed at 90°C with the WT and mutant templates (tm1–tm4). T1 and T2 indicate RNA products terminating at the main terminator and the first back-up terminator respectively. Transcription assays were performed with 46 nM RNAP, 238 nM TBP and 147 nM TFB.

To investigate the minimal termination signal in archaeal intrinsic termination leading to a pause state of elongation sufficiently long for termination to take place, we introduced point mutations into the terminator region to stepwise alter the number of consecutive T residues (Fig. 2A). The rate of termination efficiency at 90°C was determined by the amount of readthrough transcripts terminated at the first back-up terminator (T2). With five or more T residues, more than 80% of the transcription products were terminated at T1 (Fig. 2B, lanes 1 and 2). The mutations that leave four or less T residues (Fig. 2B, lanes 3–5) resulted in strongly decreased termination efficiency. The efficiency of duration of the pause at the T tract is partly determined by the availability of uridine triphosphate (UTP) in the reaction. By increasing the UTP concentration, a slight decrease of termination efficiency at all terminators can be observed, but still T1 acts as main termination site (data not shown).

### Possible formation of a hairpin structure has no effect on termination

For bacterial termination, it is well known that a GC-rich dyad symmetry element capable of stem-loop formation preceding the oligo-dT tract plays an essential role in transcriptional termination. For several pol III-transcribed genes, a palindromic sequence immediately upstream of the termination site has been described. Bogenhagen and Brown (1981) could show that these sequence elements have no influence on termination efficiency of the 5S RNA gene. In contrast, in another study it has been shown that dyad symmetry elements preceding the terminator stimulate pol III (Chu *et al*., 1997). In a thermophilic *Methanothermobacter*-derived archaeal transcription system, the presence of a sequence being capable of stem-loop formation was shown to contribute to termination efficiency (Santangelo and Reeve, 2006).

**Fig. 3:**
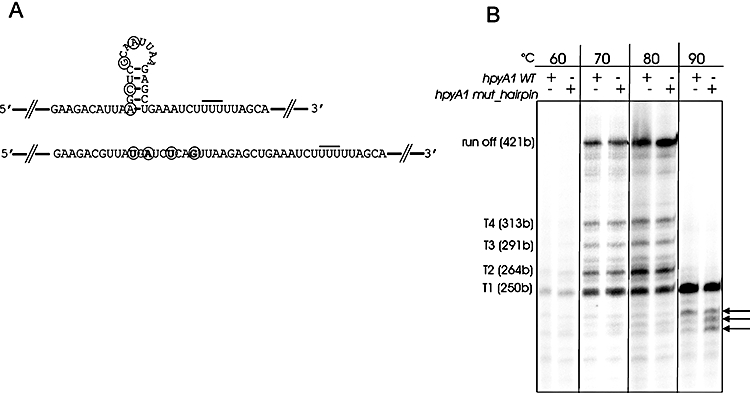
A putative hairpin structure has no effect on termination. In (A), the 3′ end of the RNA product from T1 is depicted. The palindromic sequence preceding the terminator is capable of hairpin formation. Point mutations introduced to eliminate the dyad symmetry are shown in bold and encircled in the sequence (lower panel); the mutated positions are encircled in the secondary structure. The termination site T1 is indicated by a bar on top of the consecutive U residues. *In vitro* transcription products of the linearized WT (*hpyA1* WT) and mutant templates (*hpyA1 mut_hairpin*) are shown in (B). The lengths of the RNA products are indicated on the left. Paused transcripts are indicated by arrows. *In vitro* transcription was performed at incubation temperatures as indicated above and contained 46 nM RNAP, 238 nM TBP and 147 nM TFB.

The *hpyA1* gene shows a palindromic sequence within the ORF located 6 nt upstream of the T stretch representing T1. It is unlikely that this stem loop consisting of a stem of 5 bp is stable in purified RNA at temperatures between 60°C and 90°C, but a RNA secondary structure might be formed in transcribing ternary complexes. For example, the phage λ tR2 terminator has been shown to work in a *Thermus aquaticus* system at 65°C (Naryshkina *et al*., 2006). To investigate the potential contribution of the palindromic sequence in the *hypA*1 gene to termination, we introduced single-point mutations that eliminated the dyad symmetry (Fig. 3A; template *mut_hairpin*). At 90°C, the major 250 nt RNA product from the *mut_hairpin* template was somewhat weaker than the transcript from the wild-type (WT) template (Fig. 3B, lanes 7 and 8). This result would be consistent with the findings of Chu *et al*. (1997), but may as well be an effect caused by the pausing of RNAP at the *mut_hairpin* template upstream of the first termination site (Fig. 3B, lane 8 see arrows). The high-temperature conditions under which the transcription assays were performed in this study (80–90°C) are likely to destabilize RNA secondary structures. Therefore, the temperature dependence of transcription from the WT and mutated template *mut_hairpin* was analysed also at temperatures ranging from 60°C to 80°C (60°C is the lowest incubation temperature allowing RNA synthesis in the *P. furiosus* system; Naji *et al*., 2007). At 60°C, an evaluation of termination efficiency is difficult because of the low amounts of transcript obtained after an incubation time of 10 min at this temperature. No effect of the *mut_hairpin* mutation could be observed on the transcription efficiency at 70°C and 80°C. Therefore, we conclude that the palindromic sequence preceding the T tract seems to be no general requirement for archaeal transcription termination at the *Pyrococcus* histone gene *A*.

### Competition experiments reveal that the RNAP is committed to reinitiate at the same template molecule

**Fig. 4:**
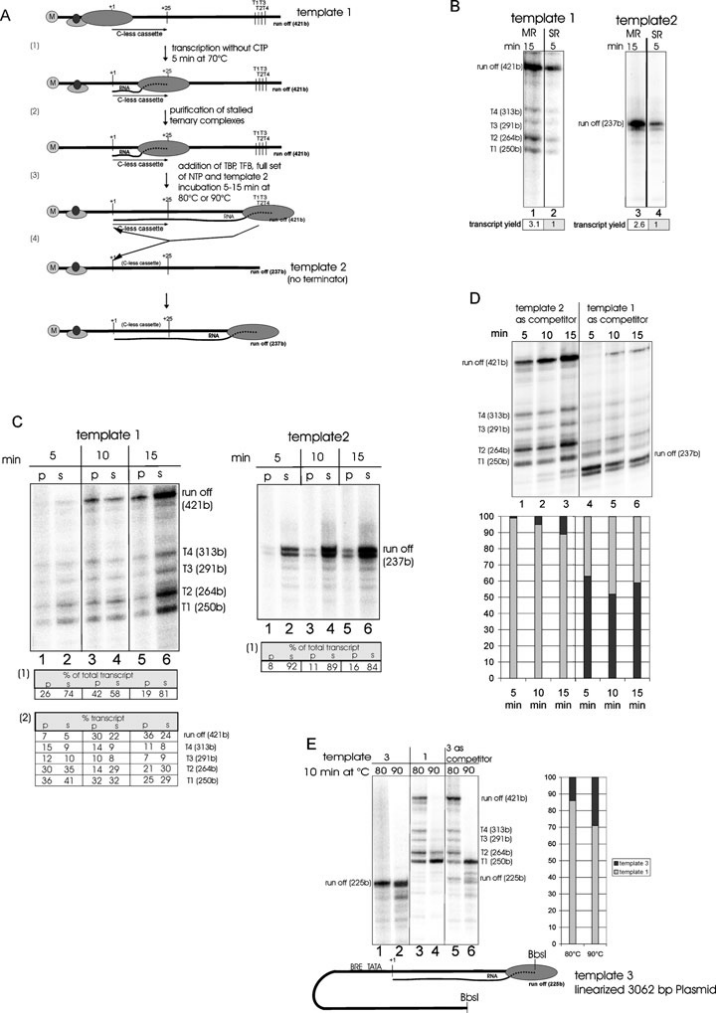
Competition experiments reveal template commitment of an archaeal RNAP. A The *hpyA1-C25* containing the termination region (template 1) was incubated in transcription reaction without CTP to obtain stable transcription complexes stalled at position +25 (1). The complexes were isolated by magnetic attraction, the supernatant was removed and the complexes were washed with 0.5% NLS to remove all promoter-bound transcription factors (2) (see Spitalny and Thomm, 2003). Then transcription buffer containing a full set of NTPs [440 μM each ATP, GTP, CTP, 2.7 μM UTP and 0.074 MBq [α-^32^P]-UTP (110 TBq mmol^−1^)], TBP and TFB (119 nM and 147 nM respectively), and competitor template (template 2) was added (3). Template 2 is a truncated version of *hpyA1-C25* not containing a terminator. It produces run-off transcripts ending 13 nucleotides before the first termination site. In the subsequent continued elongation at 80°C or 90°C (incubation time and temperature as indicated), the RNAP may choose either template to reinitiate transcription (4). For control reaction, the same procedure was used with stalled complexes on template 2 and template 1 as competitor (Fig. 4D, right panel). B. Single-round (SR) transcription compared with multiple-round transcription (MR) from the *hpyA*1 promoter at 80°C in the presence (template 1) and absence (template 2) of a linked terminator. The transcript yields were indicated below the lanes. C. The terminator delays transcript release. The amount of RNA produced by isolated complexes after the indicated incubation time (procedure see A, steps 1–3) is shown. The RNA that is polymerase-bound within the ternary complex (pellet, p) is shown in comparison with RNA that has already been released (supernatant, s). The tables below show the percentage of transcript after correction for the varying amount of radioactive UMP incorporated into RNAs of varying length. (1) shows the percentage of pelleted transcripts versus transcripts in the supernatant relative to the total of RNA (*P* + s) after 5, 10 and 15 min respectively. In (2), the percentage of transcript in each band relative to the total RNA in each lane is shown. D. The terminator directs RNAP to the same template. The experiment shown in the left panel was conducted with template 2 as competitor DNA (procedure as shown in A) and, in the experiments shown in the right panel, template 1 was used as competitor and template 2 to stall and isolate transcription complexes. Incubation time was as indicated. The diagram below demonstrates the relative amount of transcripts from template 1 (light grey) to transcripts from template 2 (dark grey). E. Template commitment at 90°C. Template 3 is a 3062 bp plasmid containing the *hypA1* gene linearized with BbsI. Template 3 was not immobilized. This terminator-less template 3 was used as competitor at 80°C and at 90°C. The diagram shows the amount of transcript from template 1 (light grey) and template 3 (dark grey) respectively. The incubation time for the reactions shown in lanes 1–6 was 10 min. The competition experiment was performed according to the scheme depicted in (A), but template 3 was used as competitor.

The limited information on archaeal termination suggests a pol III-like mechanism. It has been described (Wolffe *et al*., 1986; Kassavetis *et al*., 1989) that pol III-transcribed genes are efficiently subjected to several rounds of transcription, and the pol III transcription cycle is characterized by an increased reinitiation of pol III on the same template presumably without release of RNAP (Dieci and Sentenac, 2003). To investigate whether the archaeal RNAP is recycled from the terminator to the promoter, competition experiments between two templates were performed. Both templates contained the *hpyA1* promoter and a C-minus cassette ranging from position +1 to +25 (Fig. 4A). This construct allows stalling of RNAP at +25 by conducting transcription assays in the absence of cytidine triphosphate (CTP). Promoter-bound transcription factors can be removed by washing stalled complexes with N-lauroylsarcosine (NLS) and by purification of the immobilized ternary complexes which, in contrast to promoter-bound initiation factors, are insensitive to NLS (Spitalny and Thomm, 2003). The second template used as competitor in the experiments described in Fig. 4C is a truncated version that did not contain a terminator (template 2 in Fig. 4A) and produced a run-off transcript of 237 nt (13 nt shorter than the RNA terminated at T1). For analysis of single-round transcription, purified stalled complexes (step 2 of Fig. 4A) were chased by a full set of NTPs, but in the absence of additional TBP/TFB. Having the same promoter sequences, both templates were transcribed with similar efficiency at 80°C in single-round transcription reactions (Fig. 4B, SR). To analyse multiple rounds of transcription, stalled RNAPs were washed with transcription buffer not containing NLS, leaving TBP and TFB bound to the promoter (Spitalny and Thomm, 2003). As no free RNAP is present in these assays, newly initiated transcripts can be only formed under these conditions by RNAP molecules released from the terminator or end of the template or recycled from the terminator or the end of the DNA fragment to the promoter. Analysis of transcripts synthesized in multiple-round assays revealed that the ratio multiple-round to single-round transcripts was 3.1 for template 1 and 2.6 for template 2 (Fig. 4B).

We next analysed transcript release from both templates by stalling RNAP at position +25, followed by isolation of ternary complexes (steps 1 and 2 in Fig. 4A). Next, four NTPs but no competitor DNA were added to the stalled complexes and, after incubation times ranging from 5 to 15 min, ternary complexes were again purified, and the elongation products in ternary immobilized complexes (p) and released RNA in the supernatant (s) were analysed (Fig. 4C). As expected, transcript release was delayed at the terminator containing template (compare, e.g. lanes 1 and 2 and 3 and 4 in the left and right panel of Fig. 4C). The tables (1) below the gel panels in Fig. 4C show the total amount of transcript in the pellet obtained by magnet particle separation following step 3 (Fig. 4A) containing the ternary complexes versus the amount of released transcripts found in the supernatant. While with template 2, after 10 min, more than 80% of the run-off transcript were found in the supernatant (Fig. 4C, right panel, lanes 3 and 4), only about half of the transcribed RNA of template 1 was released after 10 min (Fig. 4C, left panel, lanes 3 and 4). But after 15 min incubation, ∼80% of RNAs were released from both templates. Table (2) below the gel panels in Fig. 4C documents the percentage of transcription products from T1, T2, T3 and T4, and of the run-off product for each lane. The numbers are corrected for the varying amount of incorporated radioactivity for each transcription product which depends upon the occurrence of uridine monophosphate (UMP) in the transcript. As expected, the T1 signal decreased while the run-off signal increased with time, indicating a slow readthrough the termination signals at 80°C. Thus, the oligo–dT tracts lead either to termination or to a slow elongation through the terminator region.

The competition experiments were first performed at 80°C as shown in Fig. 4A. Initially, transcribing polymerases were isolated while stalled at position +25. The promoter-bound transcription factors were removed by washing with 0.5% NLS (see Spitalny and Thomm, 2003). For the following elongation by RNAPs purified in ternary complexes, multiple rounds of transcription were allowed by adding a full set of NTPs and new transcription factors simultaneously with the alternative template 2. Thus, the starting conditions were the same for both competing templates. Figure 4D left panel shows the results of the competition experiments performed with template 2 as competitor DNA. Even after 15 min, when more than 80% of the transcripts formed were released and RNAP molecules were free to choose a new initiation site on both templates (inferred from the data shown in Fig. 4C), only about 10% of the total RNAs were transcribed from template 2 (Fig. 4D lane 3 and corresponding diagram below; the values were corrected for the varying amounts of UMP incorporated into the RNA products of different length). When RNAP ternary complexes were first formed on template 2 and template 1 was added as competitor, a ratio of transcripts from either template of almost 50:50 was observed already after 10 min. The finding that pre-incubation with the template lacking the terminator leads to an equal distribution of transcripts from both templates excludes the possibility that the effect is being caused by the proximity of terminator and promoter of the same DNA molecule. Our results show a clear preference for the initially transcribed template in subsequent cycles of transcription in a terminator-dependent manner, and this can be best explained by recycling of the RNAP from terminator to promoter.

To further investigate this, a template competition experiment was also performed at 90°C, using the terminator-less template 3 that showed higher stability at 90°C than the 335 nt template 2 used for the experiments shown in Fig. 4C and D. Template 3 (*hyp*A1–BbsI) consisted of a 3062 bp plasmid encoding the histone gene that was linearized by digestion with BbsI and not immobilized (Fig. 4E). Run-off transcripts from template 3 were also transcribed with high activity at 90°C (Fig. 4E, lane 2). When this template was used as competitor in step 4 of Fig. 4A at 80°C and 90°C in each case, transcription of template 1 was intrinsically favoured. At 80°C, only 14% of total RNA was transcribed from template 3 added as competitor, at 90°C only 30%. This finding shows that recycling on the first template occurs both at 80°C and 90°C in a terminator-dependent manner, and that dissociation of RNAP from the first template is increased at 90°C.

### Both terminator and associated promoter are necessary for a high transcription rate

In the pol III system (Dieci and Sentenac, 1996), facilitated recycling is dependent on the presence of a terminator sequence. As shown in Fig. 4, this is also true for facilitated reinitiation in archaea. The histone *A1* gene was predicted to have a heat shock-specific promoter (Gelfand *et al*., 2000), and this provides a possible plausible explanation for the high transcription rate at 90°C reported in this work. In fact, run-off transcription from the histone promoter on template 3 lacking the terminator occurred with similar activity at 80°C and 90°C (Fig. 5B, right panel), indicating that this promoter shows high activity at 90°C as predicted for a heat shock promoter. But the unusually strong increase, ∼2.4-fold increase of transcription at 90°C compared with 80°C observed only on a template containing both promoter and terminator (Fig. 5A, lanes 1 and 2), is strictly dependent upon the presence of the terminator.

Can the presence of this terminator also activate the expression from additional *Pyrococcus* promoters? To investigate this, the histone terminators were ligated with 163 bp of a gene segment encoding the *gdh* promoter. Run-off transcription assays from a template lacking the terminators revealed that this promoter was highly expressed at 80°C, but showed significantly lower activity at 90°C (Fig. 5B, left panel). At 80°C, the construct containing the *gdh* promoter and the histone terminators had a similar template activity as the WT histone gene (Fig. 5A, lanes 1 and 3). At 90°C, termination efficiency at T1 linked with the *gdh* promoter was as high as at the WT histone gene, but the level of total transcripts was low compared with the levels of transcripts formed at the *hpyA1* template (Fig. 5A, lanes 2 and 4). These findings suggest that the identified T tracts can act independently as termination sites irrespective of the linked promoter from which initiation was started. The facilitated reinitiation pathway proposed here for the archaeal histone promoter is highly dependent on the presence of the terminator on the same template.

### The sequences immediately downstream of the main terminator affect termination efficiency and transcription rate

As described, pausing of RNAP is a prerequisite to intrinsic termination. Palangat *et al*. (2004) showed that the sequence downstream of an internal pausing site similar to a terminator sequence affects the paused conformation of RNA pol II.

**Fig. 6:**
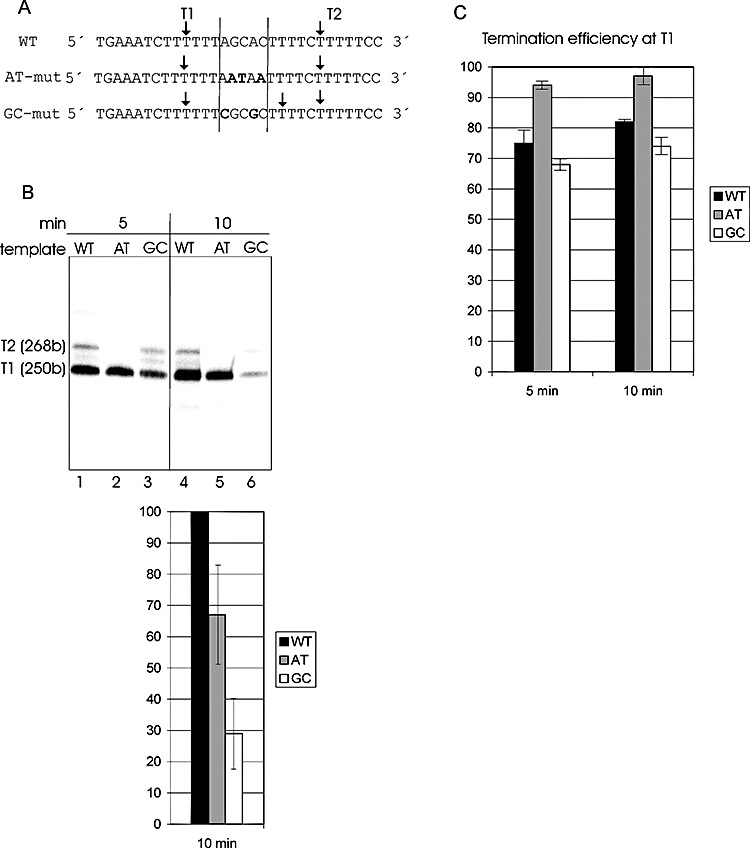
The sequences immediately downstream of the main terminator affect termination efficiency and transcription levels. A. The sequence between the main termination site (T1) and the first back-up terminator (T2) was mutated to be either AT-rich or GC-rich. The substitutions are shown in bold type. The RNA release site identified by electrophoresis of RNA products form the WT template on 20% PA sequencing gel (data not shown) are indicated by grey arrows, an additional release site induced by GC-rich downstream sequences by a black arrow. B. The RNA products obtained from the linearized WT and mutated templates containing AT- and GC-rich downstream DNA after 5 and 10 min. *In vitro* transcription was performed at 90°C and incubation times were as indicated on top of the lanes. Transcription assays were performed with 46 nM RNAP, 119 nM TBP and 147 nM TFB. The diagram below displays the amount of total transcripts transcribed from the WT template and from the different mutant templates after 10 min. C. The termination efficiency at T1 is shown for the WT and the mutant templates respectively.

To investigate whether the 3′ flanking sequence to the oligo-dT tract has any influence on termination efficiency or transcription rate in archaea, we constructed the mutated templates shown in Fig. 6A. The 5 bp between T1 and T2 were mutated to consist of only AT or GC residues respectively. *In vitro* transcription at 90°C revealed a strong effect of downstream sequences both on termination efficiency and transcription rate (Fig. 6B). The diagram in Fig. 6C shows the varying termination efficiencies at T1 relative to the overall transcript. While the termination efficiencies of the WT and GC templates do not differ significantly, the AT mutant shows a much better termination at T1.

The level of overall transcripts is strongly affected by the mutation of the sequence downstream of the termination site (Fig. 6B, diagram). The highest amount of RNA is produced by the WT DNA after 10 min of incubation (Fig. 6B, lane 4). The levels of transcripts formed at the AT mutant were reduced to around 70% compared with the WT template (Fig. 6B, diagram). The most dramatic effect was observed with the GC mutant. After 5 min, the levels of transcript were about 60% compared with the WT. Unexpectedly, after 10 min, the transcript level was reduced to ∼30% (Fig. 6B) of WT levels. This finding suggests that already after 5 min of incubation at 90°C, further RNA synthesis is significantly impaired. Our finding that two single-point mutations in the region downstream of T1 can abolish the observed activation of transcription at 90°C (Fig. 6B, lines 4 and 6) demonstrates that the sequence within the terminator region is highly critical for recycling of RNAP. Clearly, RNA degradation is expected to occur with the same rate on transcripts from all templates shown in Fig. 6A, but this degradation is compensated on the WT template by recycling of RNAP from the terminator to the promoter. Once this mechanism is impaired by the GC mutation, the rate of degradation is higher than the rate of RNA synthesis and, therefore, RNA levels are reduced to ∼30% after 10 min of incubation. GC-rich DNA immediately downstream of the terminator sequence seems to abolish facilitated reinitiation activity.

## Discussion

We selected a complete archaeal histone gene containing four consecutive downstream oligo-dT sequences as template for *in vitro* transcription experiments designed to investigate transcription termination signals and the mechanism of termination in a system operating at 90°C. The results of this study provide evidence that termination is brought about by pol III-like terminator sequences, and that the archaeal enzyme uses a pol III-like mechanism for termination.

### Termination at high temperatures and terminator signals

The *Pyrococcus* histone gene *hpyA1 has* been predicted as heat shock gene (Gelfand *et al*., 2000) and, in line with this prediction, we find unusual high levels of *in vitro* transcription of the *hpyA1* template at 90°C *in vitro* (Figs 1 and 5). *Pyrococcus* grows optimally between 90°C and 100°C, and shows slower growth at 80°C (Fiala and Stetter, 1986). Our finding that the termination efficiency at the first oligo-dT stretch (T1) *in vitro* is greatly enhanced at 90°C compared with 80°C (Fig. 1) might simply reflect the adaptation of the *Pyrococcus* transcriptional machinery to growth at high temperatures. T1 is a weak terminator at 80°C (Fig. 1), and even the three additional back-up terminators do not prevent readthrough to the end of the DNA fragment at this temperature (Fig. 1, lanes 2 and 3). Therefore, the presence of these additional terminators can reduce readthrough during growth at lower temperature, and might be an adaptation to the shallow water marine hydrothermal vent habitat of *P. furiosus* (Fiala and Stetter, 1986) that is characterized by rapidly fluctuating temperatures. The increase of the efficiency of termination with temperature could be a mechanism preventing readthrough and the expression of downstream genes upon heat shock, and thereby contribute to a specific stimulation of expression of the *Pyrococcus* histone at elevated temperatures. The specific stimulation of histone gene expression upon heat shock seems to be physiologically important, because binding of histones from hyperthermophilic archaea to DNA is likely to stabilize duplex DNA at elevated temperatures. Six or five T residues were sufficient for a high termination efficiency at 90°C, four T residues lead to a efficiency of ∼50, three T residues or less are not recognized as significant terminator signals (Fig. 2). By contrast, in the *Methanothermobacter thermoautotrophicus* (*M. t*) system, deletion of one T residue from the T6 stretch in the t_R′_ terminator resulted in a about fourfold reduced termination when termination efficiency of a construct containing this *Escherichia coli* bacteriophage terminator was assayed *in vitro* (Santangelo and Reeve, 2006). This finding suggests that the signals directing efficient termination differ slightly among archaea-like in eukaryotic pol III genes and systems from different species (Gunnery *et al*., 1999). The minimal signal sufficing for termination of the archaeal enzyme at the *Pyrococcus* histone terminator resembles most of the *Saccharomyces cerevisiae* system, which requires also five or six T residues as termination signal (Allison and Hall, 1985). A stem-loop structure upstream of the histone terminator has no effect on termination at the *hpyA1* terminator (Fig. 3). By contrast, RNA hairpins seemed to contribute to termination efficiency in the *M. t*. system at the same incubation temperature (Santangelo and Reeve, 2006). Our findings indicate great similarities of the *Pyrococcus* termination to pol III termination at the *Xenopus* 5S gene. In both systems, a stretch of T residues but no dyad symmetry sequences are required for termination (Bogenhagen and Brown, 1981).

### The mechanism of termination is pol III-like

The findings that stem-loop structures are not necessary for termination and that addition of oligonucleotides complementary to the upstream half of the RNA hairpin stem which induces RNA release in the bacterial system (Yarnell and Roberts, 1999) has no effect on archaeal termination (Santangelo and Reeve, 2006) suggest that the mechanisms of bacterial and archaeal intrinsic termination differ. In this study, several lines of evidence indicate that the mechanism of archaeal transcription is pol III-like. In the pol III system, the oligo-dT stretches in terminators induce extensive pausing of RNAP (Campbell and Setzer, 1992; Matsuzaki *et al*., 1994) without formation of a stable stem-loop structure in the RNA which causes the bacterial RNAP to pause and weakens its interactions with nascent RNA and template at rho-independent terminators (Reynolds and Chamberlin, 1992; Wang *et al*., 1997; Wilson and von Hippel, 1995; Artsimovitch and Landick, 1998). We observed a significant delay of the release of RNA from template 1 containing the set of terminators compared with template 2 lacking terminator sequences (Fig. 4C). This finding suggests that the presence of the oligo-dT residues causes also extensive pausing of the *Pyrococcus* enzyme, although pausing of *Pyrococcus* RNAP at these terminators has not been directly shown in this study.

The high *in vitro* transcription efficiency of pol III genes was shown to be mediated by a reinitiation mechanism bypassing most of the steps of the initial transcription cycle (Dieci and Sentenac, 1996). This reinitiation of pol III on the same template was dependent upon the presence of a functional terminator. We show here that the archaeal RNAP did not equally redistribute on both genes when a second template without functional terminator was added as competitor to stalled ternary complexes in multiple-round transcription assays (Fig. 4D and E). The preferred transcription of the first template indicates rapid recycling of the archaeal enzyme from the terminator to the promoter of the same template. The finding that the expression of both templates is approximately equal when the template used for formation of ternary complexes lacks a terminator suggests that pausing of RNAP at the terminator is a prerequisite for recycling of the archaeal RNAP. Our finding that only ∼10% of the competitor DNA is expressed 15 min after starting transcription from ternary complexes formed at the terminator containing template (Fig. 4D, lanes 5 and 6) suggests a mechanism involving reinitiation at the same gene without release of RNAP that was postulated to operate in the pol III system (Dieci and Sentenac, 2003).

### Effects of downstream DNA on the reinitiation mechanism

The effects of downstream DNA on termination efficiency and pausing of RNAP have been studied in the bacterial (Lee *et al*., 1990; Ederth *et al*., 2002) and human pol II system (Palangat *et al*., 2004). In the pol III system, initial (Bogenhagen and Brown, 1981) and later studies (Gunnery and Mathews, 1995; Gunnery *et al*., 1999) revealed that the sequence context around oligo-dT terminator signals modulates termination efficiency. The dinucleotide CT immediately downstream of the 3′ flank of terminators containing five T residues (T5) weakened termination efficiency, whereas an A or G residue following the T5 track increased termination (Braglia *et al*., 2005). But on terminator sequences consisting of six T residues like T1 downstream of the *hpyA1* gene analysed in this study, the weakening effect of the CT dinucleotide was lost (Braglia *et al*., 2005). In general, mutation lowering the duplex stability downstream of the T5 track increased readthrough at the pol III terminator, and thus weakened terminator efficiency (Braglia *et al*., 2005). By contrast, the mutation lowering the duplex stability immediately downstream of the *hpyA1* terminator increased termination efficiency (Fig. 6B and C, AT mutant). In the bacterial system interstrand cross-linking of the DNA duplex downstream of the terminator decreased termination efficiency, indicating that forward translocation of RNAP and melting of downstream DNA favour RNA release (Santangelo and Roberts, 2004). Considering the extreme temperature in the *Pyrococcus* system, the GC content of downstream DNA is likely to have a more important effect on DNA melting and translocation of RNAP. According to the forward translocation model, AT-rich sequences are likely to increase translocation and to reduce pausing at the terminator, GC-rich sequences probably favour pausing and inhibit downstream DNA melting and translocation of RNAP. The WT sequence downstream of *hpyA1* shows an intermediate GC content favouring overall transcript synthesis most likely by allowing the reinitiation mechanism to occur. The AT-rich mutant sequence is likely to reduce pausing of RNAP at the terminator by stimulating downstream DNA opening, and this is likely to impair reinitiation on the same template and to stimulate rapid release of RNA, and therefore the termination efficiency is predicted to be higher, but the overall transcript synthesis is predicted to be lower as observed (Fig. 6). The GC-rich downstream DNA mutant is predicted to favour RNAP pausing and to weaken downstream DNA opening and translocation. The finding that the GC-rich mutant shows after 5 min of RNA synthesis similar termination efficiency as the WT is puzzling at first sight. However, an additional weak termination site between T1 and T2 was utilized with higher efficiency by this mutant (indicated by an arrow in Fig. 6A) and, more importantly, the overall RNA levels observed after 10 min of incubation in transcription reactions were only 30% of WT levels (Fig. 6B). This finding suggests that the predicted extended pausing and the observed pausing at an additional site induced by the GC-rich sequence impair the facilitated recycling of RNAP by an unknown mechanism. Because the paused RNAP molecules are not available for reinitiation and the synthesized RNA is rapidly degraded at 90°C (Hethke *et al*., 1999), the overall levels of RNA decrease with extended incubation time as observed (Fig. 6). Our findings indicate that a subtle balance between translocation of RNAP beyond the oligo-dT stretch and pausing at the terminator is important for termination and recycling in the archaeal system. The characteristics of this *Pyrococcus* system, recycling and reduced stability of RNA at 90°C, are responsible for the unexpected and, to our knowledge, unique effects of AT-rich and GC-rich downstream sequences on termination efficiency and the level of transcripts observed.

## Experimental procedures

### DNA templates for *in vitro* transcription

Histone A1 gene (*hpyA1*) including promoter and terminator regions was amplified from *P. furiosus* genomic DNA using the primers his-a1_F 5′-GGC AAT CTA TTT GGA A**T**T CGC TCT G-3′ and his-a1_R 5′-GAT ATA CTT TAA TTT C**T**G **C**AG GCT C-3′ containing a restriction site for EcoRI and PstI respectively. The fragment was inserted between the corresponding sites of pUC19. The resulting plasmids were transformed into *E. coli* JM109, amplified and purified. They were used as linearized templates after restriction with PstI.

The templates with point mutations in the terminator region were constructed by polymerase chain reaction (PCR) using two internal primers complementary to opposite strands of the plasmid pUC19 with *hpyA1*, both containing the desired point mutations. With one of the internal primers and either the M13 F or the M13 R primer, two fragments with overlapping sequences in the region of the internal primers were produced. In a second PCR, the overlapping sequences were fused and the complete fragments were amplified with the flanking M13 primers. The sequences of the point mutations were confirmed by sequencing and are noted in the corresponding figures.

To construct the template *gdhWTPr + hpyA1_term*, the terminator region from *hpyA1* was amplified from *P. furiosus* genomic DNA using the internal primer Hisa1_mH 5′-CAA GGC ACG CA**T C**TA GAA AGA C-3′ and his-a1_R. After restriction of the PCR fragment with XbaI and PstI, the fragment was inserted between the corresponding restriction sites directly downstream of a *gdh* gene segment in pUC19 containing the *gdh* sequence from −95 to +163 from *P. furiosus.* The following steps were as described above. The mutations were confirmed by sequencing.

### *In vitro* transcription assays

*In vitro* transcription assays were performed as described previously (Hethke *et al*., 1996). A standard transcription reaction mixture of 25 μl contained 250 ng of linearized plasmid DNA, 46 nM RNAP, 238 nM or 119 nM recombinant TBP (as indicated in figure legends), 147 nM recombinant TFB, 440 μM each ATP, GTP, CTP, 2.7 μM UTP and 0.074 MBq [α-^32^P]-UTP (110 TBq mmol^−1^). The transcription buffer contained 40 mM HEPES, 0.1 mM EDTA, 1 mM DTT, 275 mM KCl and 3 mM MgCl_2_. Transcription reactions were performed 10 min at 90°C or as indicated in figures. Transcription reactions were stopped by the addition of loading buffer (98% formamide, 10 mM EDTA and 0.1% each bromphenol blue and xylene cyanol). Labelled transcripts were separated by electrophoresis on 6% polyacrylamide urea gels and visualized by phosphorimaging (FLA-5000, Fuji, Japan).

### Immobilized *in vitro* transcription assays and competition experiments

To allow pausing and isolation of RNAP on the immobilized DNA template, a C-minus cassette was introduced into the *hpyA1* gene by PCR. Two internal primers complementary to opposite strands of the *hpyA1* sequence from −6 to +23 relative to the transcription start site were used to substitute all C residues until position +25 (hpfA1-C25F 5′-CAA AAT GGA AAT GTG TTA TAA ATA AAA GG-3′, hpfA1-C25R 5′-CCT TTT ATT TAT AAC ACA TTT CCA TTT TG-3′). The plasmids were constructed and transformed into *E. coli* as described above. The templates for immobilized *in vitro* transcription reaction were produced by PCR of pUC19 containing *hpyA1* with C-minus cassette (*hpyA1-C25*) by the use of the primers M13F and M13R. M13F was 5′-modified with biotin, and the resulting PCR fragments were immobilized on streptavidin magnetic beads (Roche Applied Science) according to the protocol of the manufacturer. To isolate transcription complexes, the immobilized template *hpyA1-C25* was incubated in transcription reaction. A 25 μl of reaction contained 30 ng of immobilized template, 46 nM RNAP, 119 nM recombinant TBP, 147 nM recombinant TFB, 40 μM each ATP, GTP, 2 μM UTP and 0.074 MBq [α-^32^P]-UTP (110 TBq mmol^−1^). Buffer conditions were as listed above. Transcription reactions were performed 5 min at 70°C. Transcription complexes paused at position +25 were isolated by magnet attraction at room temperature, washed with transcription buffer containing 0.5% NLS. Washing with NLS removes promoter-bound transcription factors, but stalled RNAP is retained in ternary complexes (Spitalny and Thomm, 2003). The isolated ternary complexes were resuspended in transcription buffer and supplemented with all four nucleotides [440 μM each ATP, GTP, CTP, 2.7 μM UTP and 0.074 MBq [α-^32^P]-UTP (110 TBq mmol^−1^)], 119 nM recombinant TBP and 147 nM recombinant TFB to allow the stalled RNAPs to continue elongation (chase). Chase reaction was performed at 80°C or 90°C, and the incubation time is indicated in the corresponding figures. For single-round transcription, TBP and TFB were omitted from the chase reactions (step 3 of Fig. 4D). Multiple-round transcription in Fig. 4B was performed by washing with transcription buffer not containing NLS. These reactions contained no excess transcription factors but promoter-bound transcription factors directed multiple rounds of transcription by RNAPs isolated by steps 1 and 2 of Fig. 4A.

For competition experiments, immobilized *hpyA1-C25* templates lacking the terminator were constructed by PCR. The M13 F primer was biotinylated and the reverse primer was complementary to the region 12–33 nt before the terminator region. A total of 30 ng of this template was added to the chase reaction of the isolated transcription complexes performed as described above.
